# Composite Structural Motifs of Binding Sites for Delineating Biological Functions of Proteins

**DOI:** 10.1371/journal.pone.0031437

**Published:** 2012-02-08

**Authors:** Akira R. Kinjo, Haruki Nakamura

**Affiliations:** Institute for Protein Research, Osaka University, Suita, Osaka, Japan; Koç University, Turkey

## Abstract

Most biological processes are described as a series of interactions between proteins and other molecules, and interactions are in turn described in terms of atomic structures. To annotate protein functions as sets of interaction states at atomic resolution, and thereby to better understand the relation between protein interactions and biological functions, we conducted exhaustive all-against-all atomic structure comparisons of all known binding sites for ligands including small molecules, proteins and nucleic acids, and identified recurring elementary motifs. By integrating the elementary motifs associated with each subunit, we defined composite motifs that represent context-dependent combinations of elementary motifs. It is demonstrated that function similarity can be better inferred from composite motif similarity compared to the similarity of protein sequences or of individual binding sites. By integrating the composite motifs associated with each protein function, we define meta-composite motifs each of which is regarded as a time-independent diagrammatic representation of a biological process. It is shown that meta-composite motifs provide richer annotations of biological processes than sequence clusters. The present results serve as a basis for bridging atomic structures to higher-order biological phenomena by classification and integration of binding site structures.

## Introduction

Virtually every biological process is realized, at the atomic level, through a series of interactions between proteins and other molecules. Accordingly, most proteins, if not all, synchronously or asynchronously interact with multiple molecules ranging from single atom ions, small (non-polymer) molecules to proteins, nucleic acids and other macromolecules. The types and combinations of interactions in proteins are known to modulate their functions. For example, depending on their ligand-bound or ligand-free forms as well as interactions with corepressor or coactivator proteins, nuclear receptors can perform intricate transcriptional regulations [1]. The activity of coronavirus 3C-like proteases is controlled by dimerization through their C-terminal domain which is absent from picornavirus 3C proteases [2]. Furthermore, certain homologous proteins can catalyze completely different enzymatic reactions, namely transferase or hydrolase activities, by adopting different oligomerization states [3]. Therefore, it is important to identify not only individual interactions, but also possible combinations of the interactions that can be accommodated by a protein to fully describe its molecular functions as well as to distinguish different functions among homologous proteins.

The advance in genome sequence technologies is making it more and more imperative to develop effective techniques for inferring protein functions from sequence information. To date, the most widely used approach for protein function prediction is the annotation transfer, which is based on the assumption that protein functions are similar if their sequences are similar [4]–[6]. It has been gradually recognized, however, that such annotation transfer approaches may be unreliable in many cases [7], [8]. It has also been shown that function similarity is not a simple function of sequence similarity [9]. These observations prompt us to have more detailed examination of the determinants of protein functions.

Structural information has been proved valuable for precisely understanding protein functions [10]. Thanks to the structural genomics efforts [11], [12], we now have a great wealth of structural information available for close examination of sequence-structure-function relationships of proteins. However, when global topologies or folds of protein structures are considered, it is often even more difficult to assign a specific function to a particular fold, for some folds include an extremely diverse set of proteins with diverse functions [3], [13]. The use of structural information is not limited to finding global fold similarity and distant evolutionary relationship. In particular, physical interactions between protein molecules and their ligands observed in experimentally solved protein structures allow more direct approaches to elucidate the relationship between protein structures and functions [14], [15]. To date, there have been many methods for detecting potential ligand binding sites based on structural similarity of proteins [14], [16]–[22]. Most of these methods are targeted at predicting protein functions at the level of ligand binding and catalytic activity. There have also been many studies on protein-protein interaction interfaces to understand biological functions of proteins in cellular contexts [15], [23]–[34]. However, apart from a few works [35]–[37], most of these studies are focused on particular types of interactions *per se* and do not explicitly address how the combination of interactions with small molecules and macromolecules modulates biological function of proteins.

To understand the relationship between the patterns of interactions at atomic level and biological functions of proteins, we herein performed exhaustive all-against-all structural comparisons of binding site structures at atomic level using all structures available in the Protein Data Bank (PDB) [38], and identified recurring structural patterns of ligand binding sites to define *elementary motifs*. We then defined *composite motifs* by integrating the elementary motifs associated with individual subunits. In other words, protein subunits with the same combination of elementary motifs are said to share an identical composite motif. We then examined how such composite motifs correlated with protein functions as defined by the UniProt database [39]. It is demonstrated that the similarity between composite motifs better corresponds with the similarity between functions compared to the similarity between protein sequences or between individual binding sites. Finally, by integrating all the composite motifs associated with particular functions, we define *meta-composite motifs*. It is shown that meta-composite motifs are useful to elucidate the rich internal structures of biological processes compared to sets of homologous sequence clusters.

## Results

### Identification of elementary and composite motifs

We first generated all biological units as annotated in the PDBML [40] files, and then extracted 197,690 protein subunits which contained at least one ligand (non-polymer, protein or nucleic acid) binding site. Here, a ligand binding site of a subunit is defined as a set of atoms of the subunit that are in contact with some atoms of the ligand within 5 Å. While we do not use any pre-defined non-redundant data set based on sequence similarity, the redundancy is taken care of after clustering similar structures (see below). In this manner, the structural diversity of proteins with highly homologous or identical amino acid sequences can be preserved in the following analyses while the structural redundancy is removed.

**Figure 1:**
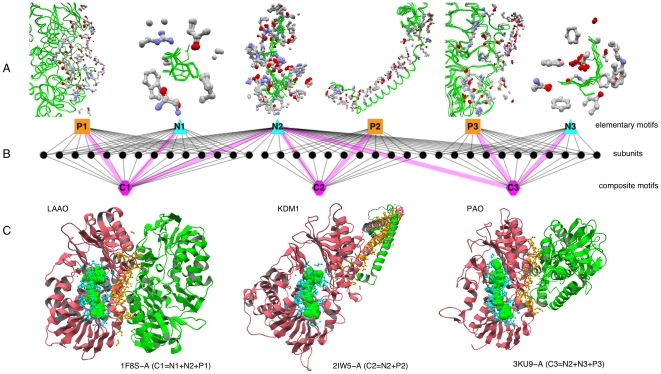
Examples of elementary and composite motifs. A: Concrete examples of elementary motifs (corresponding to B). Several binding sites belonging to each elementary motif are superimposed. The binding site atoms that constitute the elementary motif are shown in ball-and-stick representation with CPK coloring and ligands are shown in green wireframes (non-polymers) or tubes (proteins). These binding sites include subunits shown in C. Non-polymer ligands are phenylalanine and its analogs (N1), FAD (N2), and polyamines (N3). B: In this example, the combinations of 3 non-polymer binding elementary motifs (cyan triangles labeled N1, N2 and N3) and 3 protein binding elementary motif (orange rectangles labeled P1, P2 and P3) found in various protein subunits (black dots) define 3 distinct composite motifs (hexagons in magenta labeled C1, C2, and C3). Examples of each elementary motif are shown in molecular figures (A) right above the triangles or rectangles, and those of each composite motif are shown in molecular figures (C) right below the hexagons. Direct correspondence between elementary and composite motifs is indicated by thick edges in pale magenta. C: Concrete examples of composite motifs (corresponding to B). These 3 composite motifs share the same elementary motif for FAD binding (labeled N2 in B). Subunits (colored pink) containing the composite motifs (C1, C2, C3) are shown with elementary motifs in ball-and-stick representations (protein binding sites in orange, non-polymer binding sites in cyan) and with ligands in green (spacefill for non-polymers, cartoon for proteins). From left to right: L-amino acid oxidase (LAAO) from *Calloselasma rhodostoma* in homo-dimeric form (PDB ID: 1F8S [42], chain A); human lysine-specific histone demethylase 1 (KDM1) (PDB ID: 2IW5 [43], chain A); polyamine oxidase (PAO) from *Zea mays* in putative homo-dimeric form (PDB ID: 3KU9 [44], chain A, pdbx_struct_assembly.id 3). The protein figures were created using jV [75]. The network diagrams (also in Figs. 5 and 6) were created using Cytoscape [76].

All-against-all structure comparisons of 410,254 non-polymer binding sites, 346,288 protein binding sites and 20,338 nucleic acid binding sites using the GIRAF structure search and alignment program [41] followed by complete linkage clustering yielded 5,869, 7,678 and 398 clusters (with at least 10 members) of non-polymer, protein and nucleic acid binding sites, respectively. (We did not use in the following analyses small clusters with less than 10 members because some small clusters exhibited spurious similarities.) We refer to these clusters as *elementary motifs* in the following. An elementary motif can be regarded as a bundle of mutually similar atomic dispositions of binding sites (Fig. 1A). It should be noted that the elementary motifs are solely based on the binding site structures, and they do not directly include the identity of the binding partners. We have previously performed comprehensive analyses of elementary motifs [14], [15]. It was found that most elementary motifs were confined within homologous families. In some exceptional cases, motifs were shared across non-homologous families with different folds, which included motifs for metal, mononucleotide or dinucleotide binding for non-polymer binding sites [14] and coiled-coil motifs for protein binding sites [15].

The set of all elementary motifs contained in a protein subunit is called the *composite motif* of the subunit (Fig. 1B,C). Thus, two subunits sharing the same set of elementary motifs are said to have the same composite motif. In total, 5,738 composite motifs, each of which is shared by at least 10 subunits, were identified. Our hypothesis is that thus defined composite motifs exhibit good correspondence with protein functions. In the example in Fig. 1, while the three proteins (LAAO [42], KDM1 [43] and PAO [44]) share the same elementary motif (N2) for FAD binding and they share the same domain folds (FAD/NAD(P)-binding domain and FAD-linked reductases C-terminal domain [45]), their biological functions are similar but different; and these differences correspond to the differences in their composite motifs.

### Characterization of composite motifs

**Figure 2:**
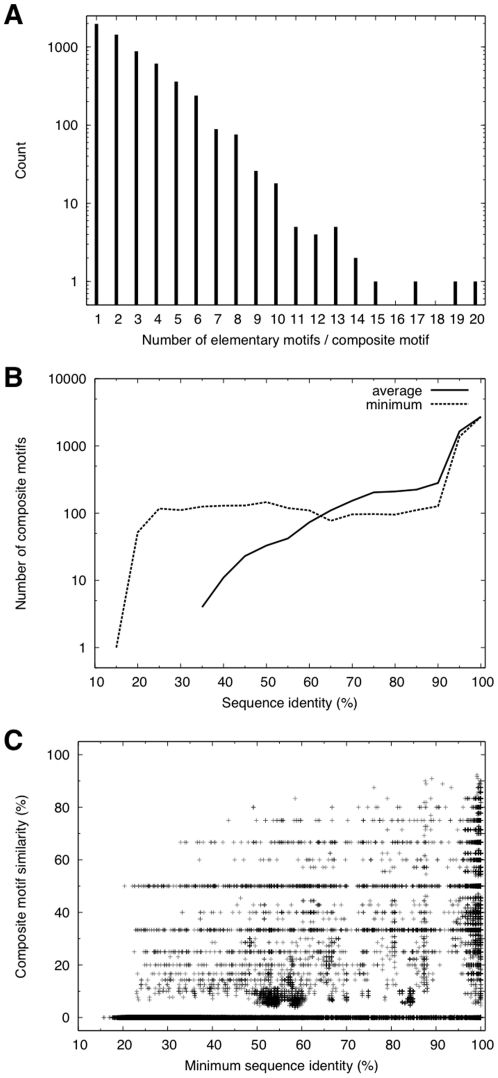
Characterization of composite motifs. A: Histogram of the number of elementary motifs comprising composite motifs. B: Histograms of the average and minimum sequence identities (%) between pairs of subunits within each composite motif. C: Composite motif similarity as a function of minimum sequence identity between pairs of composite motifs. Sequence identity between two composite motifs is defined as the sequence identity between two protein sequences, one belonging to the one motif, the other to the other motif.

The number of elementary motifs that comprise a composite motif ranges from 1 to 20 (Fig. 2A). Approximately one third of the composite motifs (1975 out of 5738) consist of only one elementary motif and more than 90% of the composite motifs are composed of less than or equal to 5 elementary motifs. The number of composite motifs appears exponentially decreasing as the number of constitutive elementary motifs increases.

To characterize the diversity of composite motifs, the average and minimum sequence identities were calculated for pairs of subunits sharing the same composite motifs (Fig. 2B). Although the majority of composite motifs are shared between close homologs on average, many of them contain distantly related subunits. In particular, 118 composite motifs were shared between subunits whose sequence similarity could not be detected by BLAST [46]. However, only three out of these 118 composite motifs consisted of more than one and at most two elementary motifs. Thus, if a composite motif consists of more than one elementary motif, it is most likely to comprise only homologous proteins.

By defining the similarity between two composite motifs as the fraction of shared elementary motifs (Eq. 4), we also examined the similarity between different composite motifs as a function of minimum sequence identity between them (Fig. 2C). While many composite motifs share no elementary motifs for the entire range of sequence identities, some do share a significant fraction of their constitutive elementary motifs in spite of weak sequence similarities. It is also noted that the composite motif similarities widely vary for high sequence identities. Thus, while each composite motif comprises homologous proteins in most cases, the converse does not hold in general so that composite motif similarity hardly correlates with sequence similarity. This observation clearly demonstrates that it is not possible to take into account the structural diversity of binding sites and their combinations by using a representative set of proteins based on sequence similarity.

### Association of composite motif similarity with function similarity

**Figure 3:**
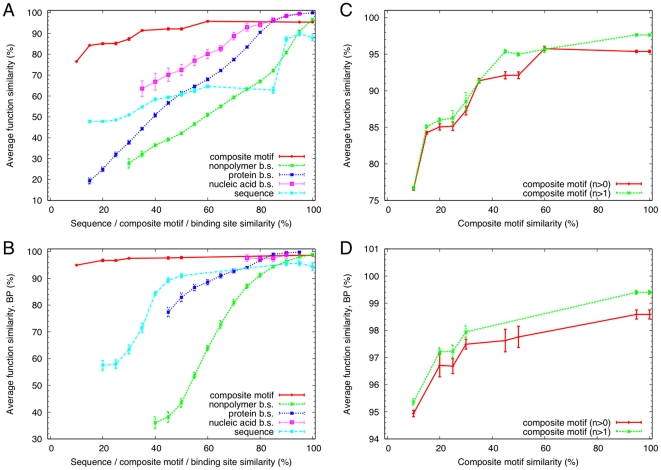
Correspondence between composite motifs and protein functions. A: Average UniProt function similarity as a function of similarity between subunits based on composite motifs, individual binding sites or sequence identity. Data points with insufficient number of samples were discarded (see Materials and Methods). Error bars indicate the standard deviation of the average function similarity based on 10 bootstrap samplings. B: Same as A, except that only the UniProt functions of the Biological process category were used. C: Composite motifs with more than one elementary motif (n
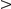
1) are compared with those with at least one elementary motif (n
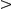
0), the latter are the same as in A. D: Same as C, except that only the UniProt functions of the Biological process category were used.

In order to study the functional relevance of the composite motifs, we next examined the association between composite motif similarity and function similarity. Here, the function of a protein is defined as a set of controlled keywords provided in UniProt [39] and the similarities for composite motifs and UniProt functions are defined by the Jaccard index (see Materials and Methods, Eq. 4). For comparison, we also checked sequence identity as well as binding site similarity (Eq. 3) as measures of subunit similarities in place of composite motif similarity (Fig. 3A). In order to reduce the bias due to the redundant data set, we randomly picked one representative from each composite or elementary motif, or sequence cluster (with 100% sequence identity cutoff) for this comparison. It is evident that the function similarity persists even for low composite motif similarities although the function similarity is not always 100% for 100% composite motif similarity. To the contrary, we can only infer high function similarities for high sequence or binding site similarities.

Since many UniProt function annotations, especially those of ligand binding activities, have been actually derived from the PDB entries, the high correlation between composite motifs and UniProt functions may appear trivial. However, the current elementary motifs that constitute composite motifs do not directly include the information of their ligands, but are solely based on their binding site structures. The bare binding site similarity does not correspond with the function similarity as strongly as the composite motif similarity. In addition, when we used only the UniProt functions under the Biological process category which are less directly related to molecular functions, we still observed the highest function similarity for a wide range of composite motif similarity compared to sequence or binding site similarities (Fig. 3B). These results demonstrate that composite motifs sharing a small fraction of elementary motifs imply more function similarity compared to bare sequence or binding site similarities.

When we examined the correspondence between composite motifs and UniProt functions excluding those composite motifs that consisted of only one elementary motif, the correspondence was found to be slightly better (Figs. 3C,D). This indicates that combinations of multiple elementary motifs may enhance accurate inference of specific protein functions.

Although the similarity between composite motifs implies similar functions, 15 composite motifs were found to be shared by completely different functions. 11, 3, and 1 of these composite motifs consisted of 1, 2, and 3 elementary motifs, respectively. 7 of them were due to improper annotations for artificially engineered proteins, to incomplete annotations in the UniProt, or to a wrong annotation in the PDB, and 3 were due to coiled-coil structures. Among the remaining 5 composite motifs, 2 composite motifs were actually found in the same dimeric complexes, and each of them consisted of only 1 elementary motif shared between remotely homologous proteins.

### Examples of composite motifs sharing the same elementary motif and fold but with different functions

**Figure 4:**
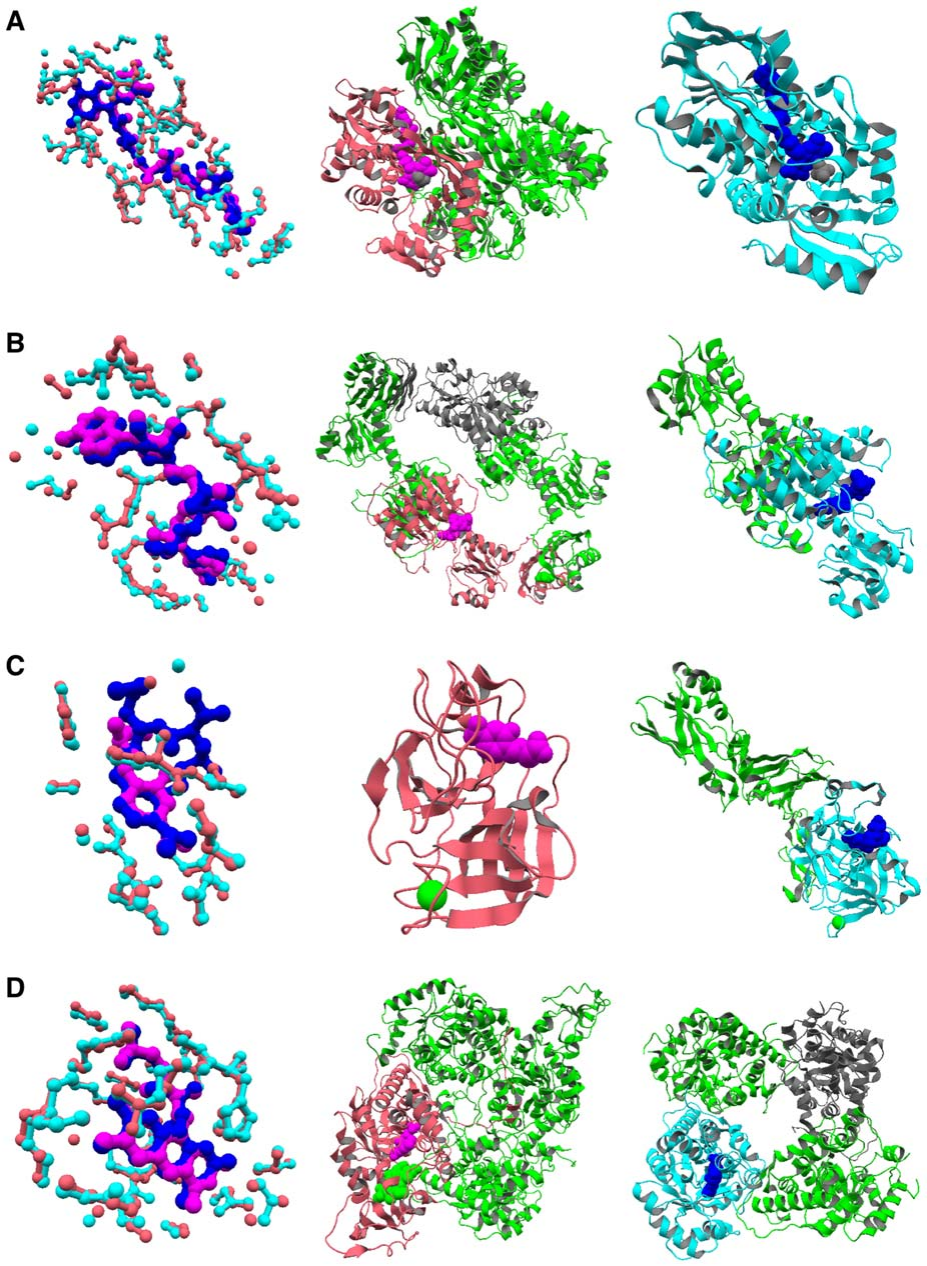
Examples of differences in composite motifs and functions. Left column: superposition of common elementary motifs (pink and cyan) and their ligands (magenta and blue). Center column: the biological unit containing the subunit with the elementary motif shown in the left column in pink, with interacting molecules (other than that in the left column) in green and non-interacting molecules in grey. Right column: the biological unit containing the subunit with the elementary motif shown in the left column in cyan, with interacting molecules (other than that in the left column) in green and non-interacting molecules in grey. A: Glycine oxidase (center) and glycerol-3-phosphate dehydrogenase (right), sharing FAD binding motif (left). B: D-3-phosphoglycerate dehydrogenase (center) and C-terminal binding protein 3 (right) sharing NAD binding motif (left). C: 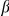-trypsin (center) and coagulation factor VII (right) sharing protease inhibitor binding motif (left). D: Cytochrome 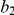 (center) and glycolate oxidase (right) sharing FMN binding motif (left).

We have already presented in Introduction an example that demonstrated different combinations of elementary motifs (i.e., composite motifs) might modulate function specificity (Fig. 1). The analysis in the previous section showed that composite motif similarity is a good indicator of function similarity. In this section, we provide several examples of proteins that share the same elementary motif and the same fold, but have different composite motifs and different functions (Fig. 4). These examples show that the difference in functions can be associated with the difference in composite motifs within the same family of proteins.

#### Glycine oxidase (GO) and glycerol-3-phosphate dehydrogenase (GlpD)

GO from *Bacillus subtilis* (PDB 1RYI [47], chain A) and GlpD from *Escherichia coli* (PDB 2QCU [48], chain A) share the same elementary motif for binding the FAD cofactor, and despite the low sequence similarity (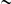
14% sequence identity), they share the same fold (FAD/NAD(P)-binding domain [45]) according to the Matras fold comparison program [49], [50] (Fig. 4A). While GO forms a homotetramer and has 3 elementary motifs for protein binding, GlpD is monomeric (however, the latter may form a dimer [48]). In addition, they have their own elementary motif for binding the respective ligands (glycolic acid, GOA, in PDB 1RYI and phosphoenolpyruvate, phosphate ion, PO4, in PDB 2R46). Thus, they have different composite motifs. In spite of the shared elementary motif for FAD binding and the shared fold, they exhibit different enzymatic activities, EC 1.4.3.19 for GO and EC 1.1.5.3 for GlpD, and function in different contexts, thiamine biosynthesis and glycerol metabolism, respectively.

#### D-3-phosphoglycerate dehydrogenase (PGDH) and C-terminal-binding protein 3 (CtBP3)

PGDH from *E. coli* (PDB 1PSD [51], chain A, EC 1.1.1.95) and CtBP3 (also called CtBP1) from rat (PDB 1HKU [52], chain A, EC 1.1.1.-) share the same elementary motif for binding the NAD cofactor and the same folds (NAD(P)-binding Rossmann-fold domain and Flavodoxin-like fold [45]) with 25% sequence identity (Fig. 4B). PGDH forms a homotetramer with one of its protein-protein interface located at its additional ACT domain [53], and is involved in L-serine biosynthesis. CtBP3, forming a homodimer or heterodimer with CtBP2, is involved in controlling the structure of the Golgi complex and acts as a corepressor targeting various transcription regulators [52]. While these proteins may catalyze very similar reactions, their biological roles are clearly different.

#### 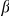-trypsin and coagulation factor VII

Bovine 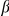-trypsin (PDB 1G3C [54], chain A, EC 3.4.21.4) and human coagulation factor VII heavy chain (PDB 1WQV [55], chain H, EC 3.4.21.21) are both serine proteases with 40% sequence identity. In these structures, they share the same elementary motif for protease inhibitors at the catalytic sites in addition to similar calcium ion binding sites although the latter do not belong to the same elementary motif (Fig. 4C). Factor VII heavy chain is in complex with its light chain counter part as well as with tissue factor, which shapes its functional form. On the other hand, 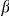-trypsin is not known to form a similar complex structure. Thus, the difference in their complex structures can be associated with the difference in their functions: digestion for 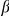-trypsin and blood coagulation for Factor VII.

#### Cytochrome 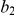 and glycolate oxidase (GOX)

Mitochondrial cytochrome 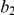, also known as L-lactate dehydrogenase, from *Saccharomyces cerevisiae* (PDB 1FCB [56], chain A, EC 1.1.2.3) and glycolate oxidase (GOX) from spinach (PDB 1AL7 [57], chain A, EC 1.1.3.15) share the TIM-barrel fold with 40% sequence identity, and have the same elementary motif for flavin mononucleotide (FMN) (Fig. 4D). Although they form roughly equivalent homotetrameric complexes, the number of interacting subunits are different: a subunit of cytochrome 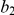 interacts with all 3 other subunits whereas that of GOX interacts with only 2 out of 3 other subunits. In addition, cytochrome 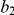 also has an elementary motif for heme binding in its additional heme-binding domain which is utilized for transferring electrons to cytochrome 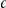 following oxidation of lactate [56]; such function is absent from GOX.

### Meta-composite motifs for annotating functions

**Figure 5:**
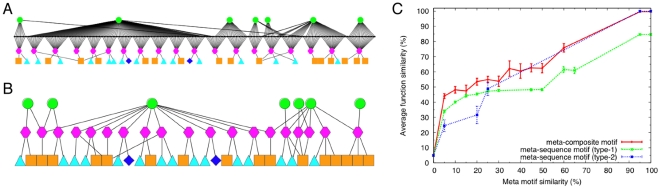
Meta-composite motifs. A: A meta-composite motif is defined as a set of all composite motifs (hexagons in magenta) associated with particular UniProt functions (green circles). The associations are defined through individual protein subunits (black dots); see text for the detailed definitions. Each composite motifs are associated with elementary motifs for non-polymer (triangles in cyan), protein (rectangles in orange), or nucleic acid (diamonds in blue) binding sites (c.f. Fig. 1). B: A simplified representation of the diagram shown in A. C: Average function similarity as a function of meta-composite motif similarity or meta-sequence motif (type-1 and type-2) similarity.

While each composite motif describes a particular state of a protein subunit, any biological process is realized as a series of interaction patterns. In this sense, composite motifs only represent snapshots of biological processes. To have a more integrative view of biological processes, we define *meta-composite motifs* by grouping all the composite motifs associated with particular functions (Fig. 5A,B). For 3,359 UniProt functions, 2,760 meta-composite motifs were identified. The number of composite motifs associated with meta-composite motifs ranged from 1 to 157, with the average of 2.39 (S.D 4.62). While the same UniProt function implies the same meta-composite motif by definition, the converse does not hold in general as there are more functions than meta-composite motifs. Meta-composite motifs thus allow us to understand protein functions as an ensemble of snapshots of ligand-bound states of proteins. For comparison, we analogously defined meta-sequence motifs by associating each function with corresponding sequence clusters (complete linkage). We defined two types of sequence clusters, the one (type-1 sequence cluster) is based solely on BLAST E-value cutoff of 0.05, the other (type-2 sequence cluster) is based on sequence identity cutoff of 100%. Thus, the former sequence clusters include a wide range of homologous sequences while the latter include only (almost) identical sequences.

We then compared the meta-composite motif or meta-sequence motif similarities with function similarity (Fig. 5C). It is not surprising that the function similarity appears lower for the meta-composite motif similarity than for composite motif similarity because, by definition, different meta-composite motifs always have different functions while different composite motifs may have identical functions. Although the differences are small, we can still observe that similar meta-composite motifs imply more similarity in functions than either type-1 or type-2 meta-sequence motifs (Fig. 5C).

It is also noted that the average size of meta-composite motifs (2.39
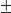
4.62) is statistically significantly greater than those of meta-sequence motifs (1.88
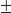
4.42 for type-1, 1.86
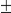
3.43 for type-2). This indicates that the composite motifs more finely dissect protein functions than the sequence clusters.

### Network structure of meta-composite motifs in biological processes

**Figure 6:**
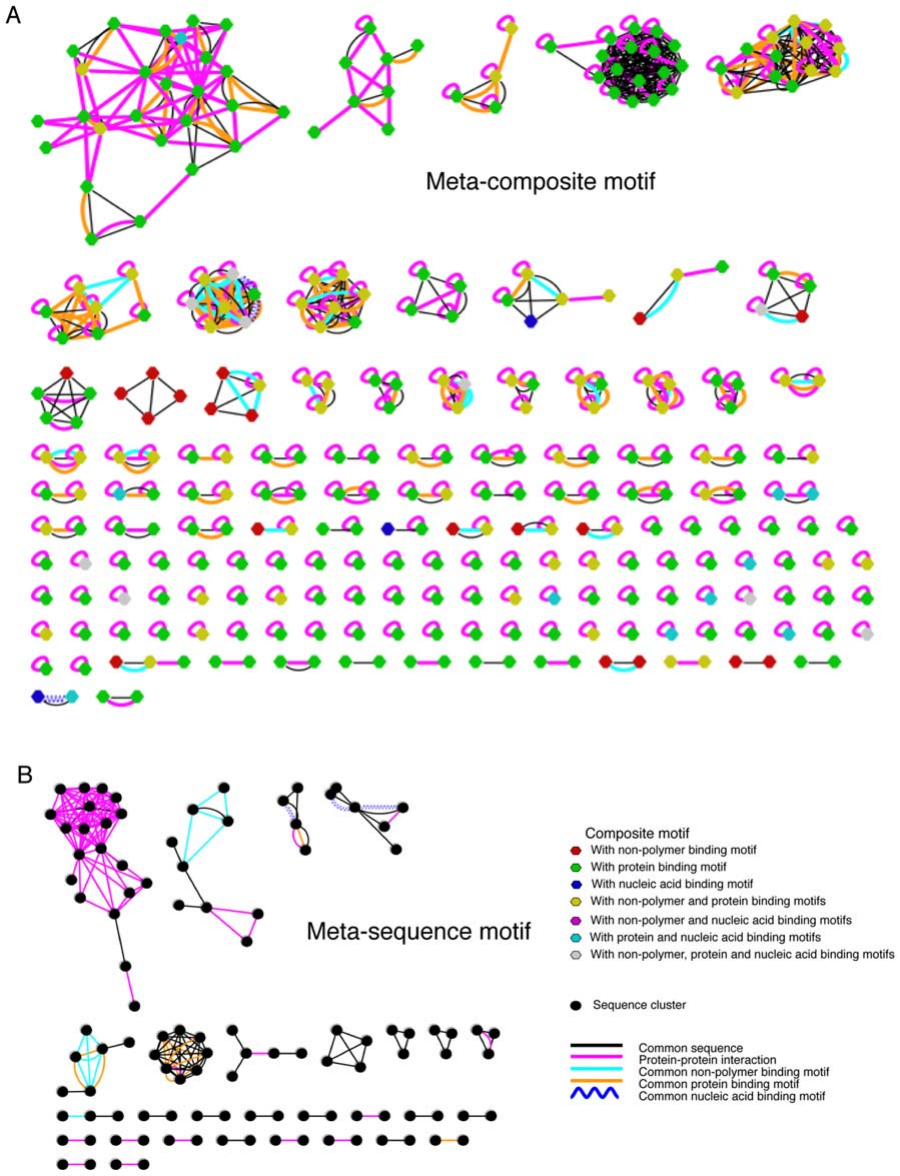
Network structure of the meta motif for biological process. Examples of a meta-composite motif (A) and a type-1 meta-sequence motif (B) for the UniProt biological process “Transcription.” A: The meta-composite motif, i.e., the set of composite motifs (colored hexagons) associated with Transcription. B: type-1 meta-sequence motif, i.e., the set of type-1 sequence clusters associated with the same keyword.

Since the meta-composite motifs are defined by grouping together all composite motifs associated with particular functions, they are more suitable for analyzing, rather than predicting, protein functions in terms of interaction states of proteins. For example, we can identify a meta-composite motif for the UniProt keyword “Transcription” (Fig. 6A), and subsequently connect the constituent composite motifs (nodes) based on relations such as common elementary motifs or common sequences. When a protein in one composite motif interacts with another protein in another (possibly the same) composite motif, an edge representing protein-protein interaction can be also drawn. In the case of composite motifs, nodes may be also characterized according to their constituent elementary motifs (i.e., interaction states). We can observe a variety of interaction states of nodes and relations between nodes. If two nodes share identical sequences, it reflects a transition between different interaction states, possibly changing their atomic structures. For example, there are PDB entries of human cellular tumor antigen p53 with or without bound DNA (e.g., PDB 1UOL [58] and 2AC0 [59]) which share the same elementary motif for zinc binding but have different composite motifs depending on the presence or absence of the elementary motif for DNA binding. Similarly, there are PDB entries of yeast RNA polymerase II with or without bound DNA/RNA in which the subunit RPB2 (e.g., PDB 1I3Q [60], chain B and 1Y1W [61], chain B) share some elementary motifs for protein binding, but other corresponding protein binding sites belong to different elementary motifs due to slight conformational changes (not shown), and an elementary motif for binding DNA is present in only one of the entries; thus these subunits identical in amino acid sequence have different composite motifs which are connected by edges of the common protein binding motifs and of the common sequence. Such description is not possible with meta-sequence motifs (Fig. 6B) because sequence similarity alone cannot discriminate different interaction states.

**Figure 7:**
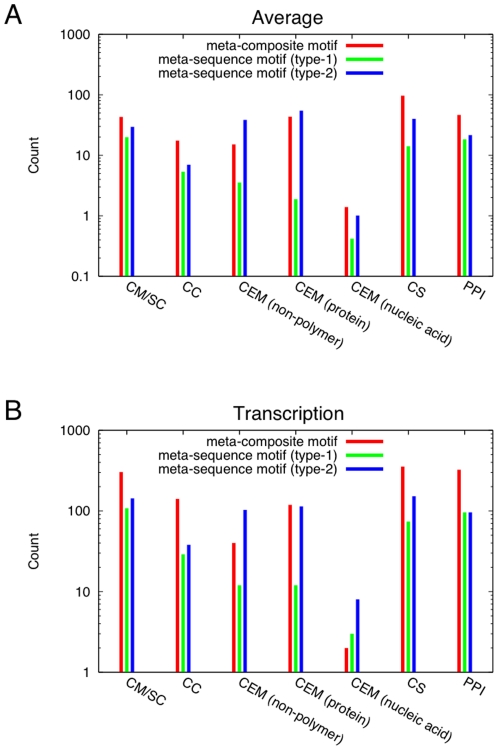
Characteristics of meta motif networks. A: Average counts of composite motifs or sequence clusters (denoted CM/SC), connected components (CC) as well as edges representing sharing of common elementary motifs (CEM) for non-polymer, protein and nucleic acid binding sites, common sequences (CS) and protein-protein interactions (PPI). B: The same counts for nodes and various edges, but only for the meta motifs for the UniProt keyword “Transcription” (corresponding to the diagrams in Fig. 6).

To evaluate the properties of networks of meta motifs more generally and more quantitatively, we identified the meta motif for each upper-most keyword in the hierarchy of the UniProt Biological process category, and compared various network characteristics of meta-composite motifs against those of meta-sequence motifs (Fig. 7). On average (Fig. 7A), meta-composite motifs include more nodes (i.e., composite motifs), more connected components, as well as more connections between nodes representing common sequences (identified by the UniProt accession) and protein-protein interactions, compared to both type-1 and type-2 meta-sequence motifs. In particular, the increased number of edges representing common sequences indicates that many identical proteins are split into different composite motifs. The same trend is also observed for a particular meta-composite motif obtained for the keyword “Transcription” (Fig. 7B). As expected, the type-1 meta-sequence motifs exhibit rather poor characteristics in most aspects because many homologs are grouped into large clusters so that differences in interaction states of proteins cannot be differentiated. While the type-2 meta-sequence motifs sometimes contain more edges for common elementary motifs, this is simply because many elementary motifs shared among homologous proteins are split into different sequence clusters irrespective of interaction states, which is reflected in the lower number of edges representing common sequences. Thus, the classification of proteins in terms of composite motifs allows us to inspect the organization of proteins involved in individual biological processes.

In summary, the observation that meta-composite motifs have more counts in nodes, connected components, common sequences and protein-protein interactions implies that meta-composite motifs discriminate the subtle differences in the interaction states or conformations of the proteins involved in the biological processes and such discrimination is not possible with meta-sequence motifs.

## Discussion

Structural classifications of proteins have been traditionally targeted at elucidating the universality of protein architectures based on the notion of structural domains. As such, it is not necessarily suitable for analyzing specific functions of particular proteins [62]. In other words, the current protein structure classifications, for a good reason, ignore the differences among protein structures within the same families or folds. The examples shown in Figs. 1 and 4 clearly show that although those proteins share the same folds, they have varied functions. Such limitations of fold classifications with respect to specific assignment of protein functions have been known for some time [13]. Recently, seemingly minute differences within protein folds are being recognized as determinants of functional specificity as exemplified by the concept of “embellishments” proposed by Orengo and coworkers [63], [64]. Although it is often assumed that domains are the units of functions, there are inherent limitations in this assumption. For example, it has been known that the combination of domains generates new functions [65], therefore it is questionable to assign one function to one domain. Furthermore, the very definition of domains is problematic as there exists no universally accepted definition of domains [66].

In this study, we avoided the complications regarding the definition of domains, and directly analyzed the atomic structures of binding sites irrespective of overall topology or homology of proteins. Nevertheless, it has been previously shown that thus identified elementary structural motifs are mostly confined within homologous protein families [14], [67], especially for protein binding sites [15]. In this sense, the classification of binding site structures are effectively not very different from the traditional protein classifications. However, by combining the elementary motifs found in individual subunit structures solved under different experimental conditions, it becomes possible to specify a particular interaction state for a particular subunit. Thus, the classification of proteins based on composite motifs differs from the traditional classification schemes in that the notion of the composite motif allows us to explicate the universality of binding site structures and the diversity of their combinations at the same time. It should be stressed that the redundancy of the current PDB is essential for identifying elementary and composite motifs since the diversity of atomic structures is not negligible even for highly homologous or identical proteins [14], [68]. In addition, different interaction states of the same protein are also useful for characterizing conformational transitions [69]–[71].

We have demonstrated that the similarity between composite motifs of proteins well indicates the similarity between their functions (Figs. 3A,B). A recent study also indicates that the integration of non-polymer and protein binding sites enhances the detection of functional specificity [37]. These results manifest the importance of the context-dependent combination of ligand binding motifs for understanding protein functions. The application of composite motifs to function prediction, however, requires some caveats. In case when we know a protein structure with bound ligands, we first need to identify the elementary motifs to which the binding sites belong. But it may not be always possible to identify all the necessary elementary motifs. In case when we only have a protein structure in its ligand-free form, it is necessary to predict its binding sites if any should exist. In this case, we need to rely on prediction based on prediction, which necessarily leads to low accuracy. While this limitation is inherent in any annotation transfer approaches, it is more stringent on the one based on composite motifs because it requires more interaction states to be solved for similar proteins. In any case, it is preferable to accumulate more structures in the PDB, not only those of completely novel folds, but also those of known folds but in new ligand-bound forms. It is worth noting that the function prediction by composite motif similarity is not based on supervised learning or parameter fitting so that the results obtained here should hold mostly valid for newly solved structures to the extent that the distribution of functionally characterized proteins in the PDB stays the same.

By grouping the composite motifs associated with particular functions, we defined meta-composite motifs. It was demonstrated that the description based on meta-composite motifs provided us with a detailed annotation of biological processes (Figs. 5,6). By describing biological processes in terms of composite motifs rather than individual structures, we can abstract the pattern of interactions so that the commonality and specificity of the interactions in different contexts, such as species or pathways, for example, can be delineated. Although there are currently some limitations in this description, such as the absence of temporal relation between composite motifs or the lack of experimental structures for some possible transient complexes, these limitations may be overcome in the future by complementing meta-composite motifs with other experimental information such as gene/protein expression or interactome analyses.

In summary, we have introduced composite motifs that well describe protein functions based on the context-dependent combinations of structural patterns of binding sites, and provide a useful means to describe the atomic details of biological processes.

## Materials and Methods

### Data set

We have used all the PDB entries as of December 29, 2010 (70,231 entries). All the biological units were generated for each entry as annotated in the PDBML files [40], except for those with icosahedral, helical, or point symmetries (mostly viruses). For the latter, only the corresponding (icosahedral, etc.) asymmetric units were used. Entries without annotated biological units were treated as they are given. Some PDB entries contain more than one biological unit all of which were used in the present study since alternative oligomeric states may (or may not) be biologically relevant. The biological units in the PDB are defined by authors and/or software (PQS [72] and/or PISA [73]). In total, 197,690 subunits in 79,826 biological units contained at least one ligand binding site.

A ligand binding site of a subunit is defined as a set of at least 10 atoms in the subunit that are in contact with some atoms of a ligand within 5 Å radius. In this study, ligands include non-polymers, proteins, and nucleic acids. The non-polymer ligands are those annotated as such in the PDBML [40] files, but water molecules were discarded. The protein ligands are those annotated as “polypeptide(L)” with at least 25 amino acid residues. The nucleic acid ligands are those annotated as “polydeoxyribonucleotide,” “polyribonucleotide” or “polydeoxyribonucleotide/polyribonucleotide hybrid.”

### Similarity between binding site structures

To compare binding site structures, we used the GIRAF structural search and alignment program [41] with some modifications to enable faster database search and flexible alignments (unpublished). GIRAF produces an atom-wise alignment for a pair of binding sites. After all-against-all comparisons of binding sites, elementary motifs were defined as complete-linkage clusters with a cutoff GIRAF score [41] of 15, as in our previous studies [14], [15]. The cutoff value was chosen so that the largest cluster did not predominate all the other clusters due to the “phase transition” of the similarity networks [14], [74]. The GIRAF score is defined as
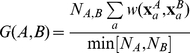
(1)where 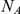 and 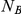 are the number of atoms of the binding sites 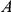 and 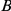 respectively, and 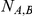 is the number of aligned atom pairs. The weight 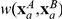 for the aligned atom pairs 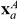 and 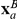 (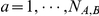) is defined as
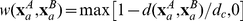
(2)where 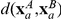 is the distance between two atoms in a superimposed coordinate system and the cutoff distance 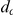 is set to 2.5 Å.

Clusters with less than 10 members were excluded in this study because structural similarity in small clusters may be coincidental. In fact, when there were protein pairs not detected by BLAST within a cluster, the fraction of such pairs was 79% on average for clusters with less than 10 members while that for clusters with at least 10 members was 36%. Although motifs shared between remote homologs or non-homologs may provide interesting examples, we expect many of them are not biologically relevant.

The raw GIRAF score largely depends on the size of binding sites. Therefore, when comparing binding site similarity with function similarity, we used a normalized similarity measure so that binding sites of varying sizes can be compared on the same scale. Let 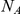, 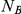 and 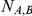 be defined as above, then the normalized similarity 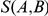 between the binding sites 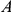 and 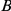 is defined as
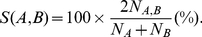
(3)


### Functions defined by UniProt keywords

For each subunit in the data set, the corresponding UniProt [39] accession identifier was obtained from the struct_ref category of the PDBML file. In total, 186,791 subunits with at least 1 ligand binding site in the PDB were annotated by UniProt. For thus identified UniProt entries, their keywords were extracted. The UniProt keywords are a set of controlled vocabulary to describe the properties of proteins and they are organized in a hierarchical order. In most cases, these keywords are manually assigned by curators, hence they are expected to be more reliable. This is in contrast to the Gene Ontology annotations (http://geneontology.org) for the PDB which are mostly automatically annotated and are likely to contain a large number of erroneous annotations.

For each subunit, all the keywords annotated either explicitly or implicitly via the keyword hierarchy, were extracted except for those belonging to the Technical term, Disease, or Domain categories. We define the function of a subunit as the set of the UniProt keywords associated with it. In other words, two subunits whose associated sets of keywords are exactly identical are defined to have the same function. In total, 7,991 UniProt functions were defined. The similarity between two UniProt functions are defined by the Jaccard index between the sets of keywords associated with the functions (see below, Eq. 4).

### Similarity between two sets

The similarity measures for composite motifs, functions or meta motifs are based on comparison between two sets. Given the sets 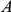 and 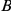, their similarity is defined by the Jaccard index 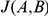:
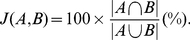
(4)


For a given composite motif, function, meta-composite motif or meta-sequence motif, the set consists of elementary motifs, UniProt keywords, composite motifs, or sequence clusters, respectively.

### Sequence clusters

To define meta-sequence motifs, complete-linkage clustering was applied to the result of an all-against-all BLAST [46] comparison with two different criteria. In one case, all pairs of sequences in a cluster must have BLAST E-value of at most 0.05. This resulted in 3,327 clusters with at least 10 members. These clusters are referred to as type-1 sequence clusters. In the other case, all pairs of sequences in a cluster must have 100% sequence identity as well as E-value of at most 0.05. This resulted in 4,594 clusters with at least 10 members, which are referred to as type-2 sequence clusters. When BLAST produces more than one alignment for a pair of sequences, the alignments were integrated into one alignment as long as they were mutually consistent.

### Comparison between motif similarity and function similarity

Although we did not use any representative set for defining elementary and composite motifs based on sequence similarity, we did use representatives of motifs and sequences when their similarities were compared with function similarity (c.f., Figs. 3 and 5C) in order to reduce the bias due to different sizes of clusters. For composite motifs, a representative was randomly selected from each composite motif. For binding sites, a representative was randomly selected from each elementary motif. For protein sequences, a representative was randomly selected from each type-2 sequence cluster. Average function similarities for a given range of motif, binding site or sequence similarity (Fig. 3) were calculated for 10 sets of randomly selected representatives and the standard deviations of the average function similarity are shown as error bars. Only those points with at least 500 (50 for nucleic acid binding sites) samples on average are shown in Figs. 3A,B.

For meta-composite and meta-sequence motifs, 50% of the all observed pairs of meta motifs were randomly selected and the average function similarity was calculated. This procedure was iterated 10 times, and data points with at least 10 samples are reported with the standard deviation of the average values in Fig. 5C.

We have confirmed that selecting different random sets of representatives in all the above cases did not alter the results significantly.

### Downloadable data

The results of all-against-all comparison of binding sites and classifications are made available for download at http://pdbj.org/giraf/cmotif/.
